# The Atlantic Monthly

**Published:** 1872-05

**Authors:** 


### The Atlantic Monthly, Volume 30th.

The publishers of The Atlantic announce that Hawthorne's Romance, SEPTIMUS FELTON, will be concluded in August. THE POET AT THE BREAKFAST TABLE, THE COMEDY OF TERRORS, and THE LIFE OF JEFFERSON, will be continued throughout the remainder of the year. The next volume, beginning with the July number, will contain A paper of peculiar interest on an obscure phase of Post-Rebellion history, by ADVOCATE GENERAL BOLLES; A story in three Chapters, By T. B. ALDRICH; Several literary papers by E. C. STEDMAN, in the vein of his article on "Tennyson and Theocritus;" A chapter of early Jesuit adventure in New York State by FRANCIS PARKMAN; Stories, by J. W. DEFOREST; Two short romances, by H. JAMES, Jr.; Sketches of Southwestern Life, by WM. M. BAKER; Siamese stories, by MRS. LEONOWENS: A paper on Mythology, by JOHN FISKE; Poems, by J. T. TROWBRIDGE, JOHN G. WHITTIER, J. R. LOWELL, etc., with many other attractive articles on various subjects.

